# Challenging management of multifocal upper extremity fractures in a polytrauma setting: A case report

**DOI:** 10.1016/j.ijscr.2024.110777

**Published:** 2024-12-26

**Authors:** Florensius Ginting, Mohammad Zaim Chilmi

**Affiliations:** a Department of Orthopedics and Traumatology, Faculty of Medicine, Universitas Airlangga, East Java, Surabaya, Indonesia; b Department Orthopedics and Traumatology, Dr. Soetomo General Academic Hospital, East Java, Surabaya, Indonesia

**Keywords:** Multiple fracture, Upper extremity, Polytrauma, Traffic Accident

## Abstract

**Introduction and importance:**

Multifocal fractures in a single upper extremity represent a significant clinical challenge, often resulting from high-energy impacts such as motor vehicle accidents or severe falls. These injuries require complex, multifaceted approaches in management, spanning initial acute care to long-term rehabilitation. This paper examines the complexities of diagnosing, treating, and rehabilitating multifocal upper extremity fractures, highlighting the importance of timely intervention and a multidisciplinary approach to maximize functional recovery, minimize long-term disability and the prognosis.

**Case presentation:**

A 25-year-old male was admitted to the emergency department after being struck by a vehicle, sustaining multiple fractures in his right upper extremity, including the scapula, proximal humerus, radius, and ulna. Initial management involved stabilizing the fractures with external fixation and performing a craniotomy for an associated epidural hematoma. After stabilization, definitive surgical repairs were performed, including ORIF for the humerus and radius ulna, followed by rehabilitation to address functional deficits.

**Clinical discussion:**

The management of multiple fractures in a single upper extremity requires timely surgical interventions, such as ORIF, to stabilize complex fracture patterns. Effective postoperative rehabilitation is essential for recovery and depends heavily on patient compliance and comprehensive care. Although advances in surgical techniques have improved outcomes, challenges remain in reducing soft tissue damage and preventing long-term complications like joint stiffness and chronic pain.

**Conclusion:**

Multiple fractures in a single upper extremity usually result from high-energy trauma, necessitating comprehensive management strategies.

## Highlights

• Multiple fractures in one extremity depend on injury mechanism and disease
• Preoperative assessment of nerve injuries in polytrauma cases can be very difficult
• The double radioulnar joint sacrifice procedure results in poor outcomes
• Complications following multifocal upper extremity fractures can be significant

## 1 Introduction and importance

Multiple fractures within a single upper extremity are a complex and rare injury pattern, often associated with significant trauma, such as motor vehicle accidents or falls from a height. These injuries, when occurring in the context of polytrauma, represent a considerable challenge in both diagnosis and management. The involvement of different anatomical regions of the upper extremity—including the clavicle, scapula, humerus, radius, and ulna—requires careful consideration to optimize both immediate stabilization and long-term functional recovery.

Data from several studies illustrate the prevalence and patterns of multifocal upper limb fractures. A study by Broadbent et al. showed that these fractures, though rare, constitute about 1.3 % of all upper limb fractures [1]. The majority of these cases involved older adults, particularly women, where falls from standing height were the leading cause. The most common combinations involved fractures of the distal radius and proximal humerus, and a large portion of these injuries occurred in osteoporotic bones.

The complexity of treating multiple upper extremity fractures lies in the need for a multidisciplinary approach. Polytrauma patients often present with other life-threatening injuries, making it necessary to prioritize fracture management based on the patient's overall condition. According to Abutalib et al., missed fractures are a common risk when only the most obvious injuries are treated initially [2]. Comprehensive assessment, including radiographic evaluation of the entire limb, is essential to avoid delayed diagnosis and complications.

Recent data suggests that multifocal fractures in the upper limb, particularly in polytrauma patients, correlate with longer hospital stays, increased disability, and more complicated recovery processes. Studies show that these fractures significantly impact the patient's functional outcome, with the Disabilities of the Arm, Shoulder, and Hand (DASH) score often being used to assess disability [1,3]. Management strategies include both early stabilization and definitive surgical intervention, with a focus on minimizing long-term.

## 2 Case presentation

A 25-year-old male arrived at the emergency department, complaining of pain and an inability to move his right arm after being struck by a truck on the right side during a motorcycle accident. At the time of impact, his arm was bracing for the collision. The patient had a history of a right forearm fracture that had been treated with plate fixation five years ago, with the plate removed one year later.

**Fig. 1:**
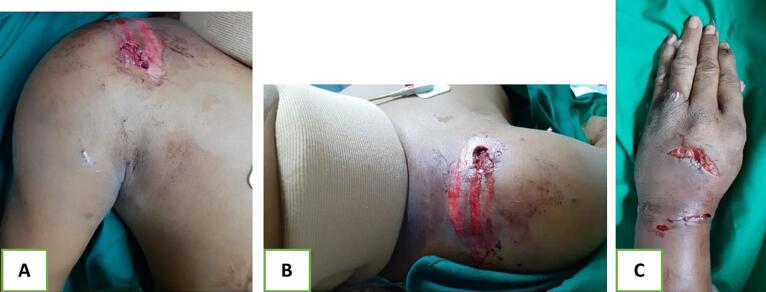
Clinical photographs of right upper extremity showing wounds and deformities; (a) shoulder anterior view, (b) superior view, and (c) posterior wrist.

During the primary survey, the patient was found wearing a soft cervical collar, displaying hypotension, tachycardia, and decreased consciousness (GCS: E2V3M4). The patient also had anisocoric pupils, along with deformities and injuries to the right shoulder, forearm, and wrist. Open wounds were noted on the right shoulder and wrist. After resuscitation, the patient's hemodynamic stabilized. The right shoulder region exhibited a 4 × 2 cm open wound with exposed muscle, and there was a 3 × 1 cm open wound with exposed bone on the wrist. Scars were present on the proximal volar and dorsal sides around the elbow (Fig. 1). There was difficulty evaluating motor and sensory functions in the affected areas. Swelling and wounds were found in the head and face area.

**Fig. 2:**
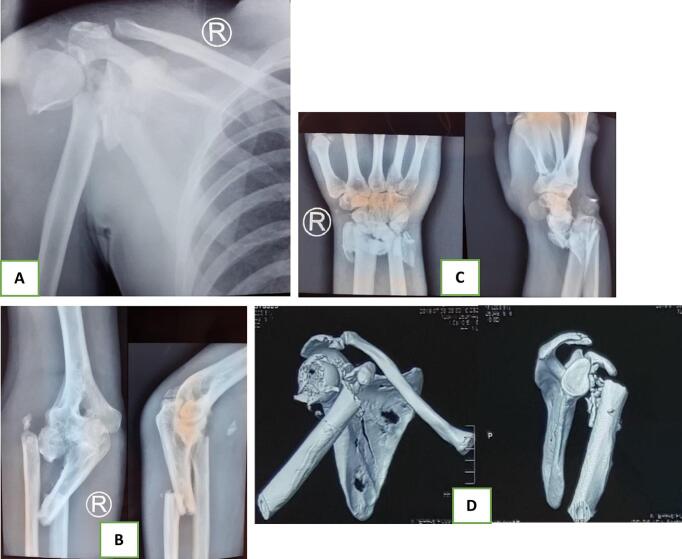
Radiographic series showing multiple fractures and dislocations of the right upper extremity; (A) shoulder anteroposterior, (B) elbow anteroposterior and lateral, (C) wrist anteroposterior and lateral, and (D) shoulder CT scan.

In laboratory tests, all parameters were normal except for leukocytosis, which was observed at 12,110/μL. Radiological imaging revealed a scapular body fracture, a displaced proximal humeral fracture (Neer 3-part), fractures of the neck of the radius and the shaft of the ulna accompanied by osteoarthritis of the elbow, and a displaced comminuted fracture of the distal radius and ulna (Frykman VIII). A CT scan was performed to clearly visualize the configuration of the proximal humerus (Fig. 2). All fractures were classified as open and categorized under Gustilo Anderson Grade 3 A. A head CT scan also revealed an epidural hematoma and diffuse axonal injury.

**Fig. 3:**
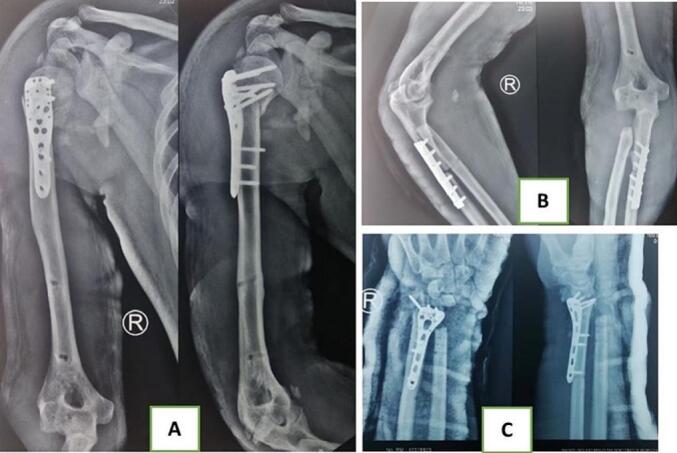
Postoperative radiographs after definitive surgical intervention; (A) humerus anteroposterior and lateral, (B) forearm anteroposterior and lateral, and (C) wrist anteroposterior and lateral.

The patient underwent emergency surgery in the operating room, which included a craniotomy for hematoma evacuation, debridement, trans-elbow and wrist external fixation as spanning joints, intramedullary pinning of the ulna, and immobilization of the proximal humerus with a coaptation splint. Wound care and regular evaluations of infection markers were performed. Three weeks later, definitive surgeries were carried out, including Open Reduction Internal Fixation (ORIF) with a proximal humeral locking plate for the right proximal humerus, ORIF plating of the ulna shaft with a small Dynamic Compression Plate (DCP), excision of the right radial head, ORIF plating of the distal radius using a Variable Angle distal radius locking plate, and a Distal Ulna Resection/Stabilization (DURS) procedure (Fig. 3). As the patient's consciousness improved, a neurological evaluation of the posterior interosseous nerve confirmed the presence of palsy; the patient was unable to extend his thumb. No other nerve injuries were found.

**Fig. 4:**
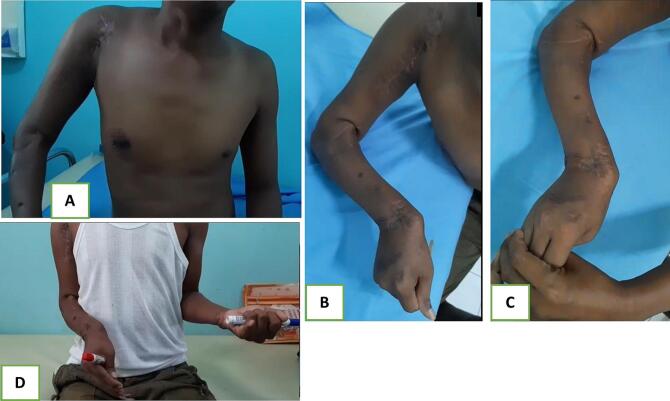
Clinical photographs and range of movement examination; (A) shoulder abduction, (B) flexion contracture elbow, (C) passive wrist extension, (D) no supination.

**Fig. 5:**
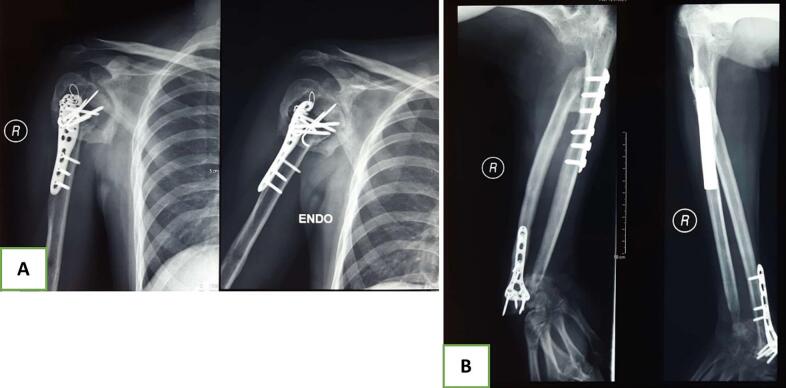
Radiological evaluation 7 months post-operative; (A) shoulder anteroposterior with internal and external rotation and (B) forearm anteroposterior and lateral.

Several weeks after visiting the outpatient clinic, the surgical wound had healed well. However, the patient was lost to follow-up due to non-compliance. Seven months post-operation, the patient returned to the clinic, complaining of restricted movement in the shoulder, elbow, and wrist. Range of motion limitations were observed in the patient's right upper extremity, with a DASH score of 78.3 (indicating extreme disability). The patient exhibits significantly restricted range of motion in the right upper extremity. The shoulder's movement is limited to 50° in abduction, 40° in flexion, and rotations are constrained to 30° internally and 20° externally. The elbow shows 75° of flexion with no extension, and rotations are restricted with pronation fixed at 60° and no supination. The wrist's flexion is reduced to 30° and passive extension to 20°, with ulnar deviation at 50° and no radial deviation (Fig. 4). These limitations severely impact the functional use of the patient's right arm. Radiological evaluations revealed avascular necrosis of the proximal humerus, union of the ulnar shaft, proximal radioulnar synostosis, malunion of the distal radius, and subluxation of the wrist joint (Fig. 5).

## 3 Discussion

Multifocal fractures in a single upper extremity, particularly when associated with polytrauma, represent a complex injury scenario requiring comprehensive management. The incidence of these injuries is notable in both high-energy trauma, such as motor vehicle accidents or falls from significant heights, and low-energy trauma in the elderly population suffering from osteopenia bones [1,[3], [4], [5]]. Studies have shown that road traffic accidents (RTAs) are the leading cause of extremity fractures, accounting for over 70 % of cases in developing countries [5]. Fracture combinations in a single upper extremity can include various anatomical locations like the proximal humerus, distal radius, and ulna, with common pairings such as fractures of the distal radius and proximal humerus [1,3,6]. Mechanisms of injury often involve significant rotational or axial forces applied to the arm during trauma, leading to complex patterns of fractures [7]. These multifocal fractures are frequently seen in polytrauma cases, where concurrent life-threatening injuries such as head trauma or thoracic injuries may complicate the patient's condition [4]. Early management focuses on the stabilization of fractures using external fixation, debridement of open wounds, and close monitoring for infection, particularly in open fractures classified under the Gustilo-Anderson system [8]. In this case, an unusual combination and configuration of fractures were found, potentially caused by a history of pre-existing fractures and related complications. Early surgical intervention is critical in reducing complications and restoring function.

In the management of multifocal fractures in a single upper extremity, treatment choices must be carefully tailored, particularly when associated with polytrauma. Initial treatment typically involves temporary stabilization, often through external fixation or splinting, to address the immediate risk of further injury or instability. This is especially crucial in polytrauma cases, where prioritizing life-threatening injuries takes precedence over orthopaedic interventions. The timing of definitive surgery, such as Open Reduction and Internal Fixation (ORIF), must consider the patient's overall condition, hemodynamic stability, and any concurrent injuries to vital organs [1,8].

Soft tissue management is another key consideration, particularly in cases with open fractures. Proper debridement and wound care are necessary to minimize infection risk, especially in Gustilo-Anderson grade III fractures. In cases of severe soft tissue damage, early and aggressive debridement, followed by soft tissue coverage using skin grafts or flaps, may be required to facilitate proper healing and prevent wound complications [7,8]. Management of the soft tissues is critical, as failure to address these injuries adequately can lead to deep infections, delayed union, or even chronic osteomyelitis.

Complications following multifocal upper extremity fractures can be significant. Common complications include non-union, malunion, avascular necrosis, and nerve palsies, particularly when fractures involve critical anatomical structures like the humeral head or distal radius [6,7]. Additionally, in polytrauma patients, prolonged immobilization or delayed surgical intervention due to prioritization of other life-threatening conditions can increase the risk of joint stiffness and functional impairment. Furthermore, if fractures are treated with external fixation alone for extended periods, it may compromise joint mobility and lead to issues such as synostosis or complex regional pain syndrome (CRPS) [4]. Close monitoring and timely follow-up are essential to detect and manage these complications early, ensuring optimal functional recovery.

In cases of comminuted distal ulna fractures, performing Distal Ulnar Resection/Stabilization (DURS) is often indicated when there is damage to the distal radioulnar joint (DRUJ), leading to pain, instability, or degenerative changes that make joint preservation impossible. DURS can alleviate ulnar-sided wrist pain and restore functional motion, particularly in pronation and supination, which are often severely limited in such fractures. This procedure is particularly advantageous in comminuted fractures where traditional fixation methods may not restore stability or joint congruence. However, it requires careful consideration of soft tissue management and the risk of ulnar stump instability, which may necessitate additional techniques like dorsal capsuloplasty or extensor carpi ulnaris (ECU) tendon stabilization to prevent long-term complications [9].

Radial head excision is often considered for fractures of the radius neck when severe comminution precludes stable fixation. This procedure can decrease immediate post-operative complications and promote quicker functional recovery. However, it risks long-term instability and altered joint mechanics, particularly if there are associated injuries like ligament tears. A case was reported with a problem involving a die-punch injury, and a Monteggia fracture described as Essex-Lopresti, with the radial head and distal radius still reconstructable using internal and external fixation, yielding satisfactory functional outcomes [7]. Careful consideration is needed to balance the benefits of pain relief and functional improvement against potential long-term joint issues [10]. Excising both the proximal and distal radioulnar joints will significantly increase the risk of instability in the radioulnar joint.

The combination of these fractures is extremely rare, making the ideal management still debatable. Additionally, there is limited literature on guidelines for managing multiple fractures in the upper extremity. The next steps in the management of this case are to restore shoulder function with arthroplasty, reconstruct the wrist, and perform a Jones transfer for drop hand. This risk is further exacerbated by the presence of posterior interosseous nerve (PIN) lesions, leading to complications such as radioulnar synostosis and subluxation of the wrist joint This case report has been reported in accordance with the Surgical Case Report (SCARE) 2023 Criteria [11].

## 4 Conclusion

Multiple fractures in a single upper extremity typically result from high-energy trauma that exerts significant forces on the limb, requiring comprehensive management strategies. Effective treatment involves timely surgical intervention, notably Open Reduction Internal Fixation (ORIF), coupled with careful post-operative care to restore function and prevent complications. Prognosis depends on the injury's severity, the promptness of medical response, and adherence to rehabilitation. Although advanced surgical techniques have enhanced outcomes, the complexity of such injuries can still lead to varying degrees of long-term functional impairment. A multidisciplinary approach is essential to maximize recovery and improve the patient's quality of life.

## Informed consent

Appropriate consent was obtained from all individual participants included in the study.

## Ethical approval

Regarding to the observational study of outcome in our case report, the ethical approval was waived by an institutional review board. However, the copies of informed consent are available for review by the Editor-in-Chief of this journal on request.

## Guarantor

Mohammad Zaim Chilmi.

## Research Registration Number

Not applicable.

## Funding statement

N/A.

## Funding

None.

## Author contribution

Florensius Ginting involved in performing surgical technique, conceptualization, investigation, project administration, resources, writing-original draft, writing-review & editing.

Mohammad Zaim Chilmi involved in performing surgical technique, formal analysis, supervision, validation, visualization, writing-review & editing.

## Conflict of interest statement

The authors have no conflicts of interest to disclose.
